# Research Ethics Review in Humanitarian Contexts: The Experience of the Independent Ethics Review Board of Médecins Sans Frontières

**DOI:** 10.1371/journal.pmed.1000115

**Published:** 2009-07-28

**Authors:** Doris Schopper, Ross Upshur, Francine Matthys, Jerome Amir Singh, Sunita Sheel Bandewar, Aasim Ahmad, Els van Dongen

**Affiliations:** 1 Ethics Review Board, Médecins Sans Frontières, Geneva, Switzerland; 2 University of Toronto Joint Centre for Bioethics, Toronto, Ontario, Canada; 3 Unit of Epidemiology and Disease Control, Department of Public Health, Institute of Tropical Medicine, Antwerp, Belgium; 4 Bioethics and Health Law Programme at the Center for the AIDS Programme of Research in South Africa (CAPRISA), Durban, KwaZulu-Natal, South Africa; 5 Research Program on Community Engagement Global Case Studies at Program on Ethics and Commercialization, McLaughlin-Rotman Centre for Global Health, University of Toronto, Toronto, Ontario, Canada; 6 The Kidney Centre, Karachi, Pakistan; 7 Aga Kahn University, Karachi, Pakistan; 8 University of Amsterdam, Amsterdam, The Netherlands

## Abstract

Doris Schopper and colleagues describe the functioning of the Médecins Sans Frontières independent ethics review board and the framework used for ethics review, and discuss challenging ethical issues encountered by the board since its inception.

**Summary Points**

- In 2001, the international humanitarian aid organisation Médecins Sans Frontières decided to institute an independent ethics review board to ensure that the increasing amount of operational and clinical research it undertakes is scientifically valid and ethical.
- This article describes the functioning of this ethics review board and the challenging ethical issues that it has discussed since its inception.

## Introduction

Médecins Sans Frontières (MSF) is an international humanitarian aid organisation that provides emergency medical assistance to populations in danger in more than 80 countries. In its work, MSF is often confronted with situations for which effective and feasible interventions are lacking. As a result, over the past few years, MSF has expanded its research activities [1]–[4]. But although MSF often works in close collaboration with scientific institutes and ministries of health that have their own ethical review mechanisms to oversee research, MSF as a humanitarian organisation has concerns that are distinct from those of academic institutions and wants to endorse with confidence any research that takes place under its name [5]–[7]. Furthermore, not all countries in which MSF works have ethics committees, some local ethics committees may not have the resources to function optimally [8]–[12], and the local or national government is not always a guarantor for the well-being of its population. For these reasons, in 2001 MSF decided to institute its own ethics review board (ERB).

### Board Composition and Function

Currently, the ERB is composed of seven members with an understanding of humanitarian and non-governmental organisation realities. The mix of members ensures good geographic (Africa, Asia, Europe, North America) and professional (medicine, public health, law, anthropology, bioethics) variety [13],[14]. To avoid conflicts of interest and to ensure independence, members cannot have a working relationship with MSF or be a member of the board of an operational centre during their tenures. Working procedures are defined in the terms of reference of the ERB [15]. Briefly, reviews are coordinated by the chair, comments are provided electronically by the board members, and discussions on divergent views mainly happen through e-mail exchange. After the ERB makes its final decision, the medical director of the MSF section concerned is responsible for ensuring that the research is implemented as approved by the ERB; the ERB is not accountable for any research carried out against its advice. Every 18 months ERB members meet in person with the MSF medical directors to discuss ethical issues that were problematic in their reviews and to make general recommendations on how to address those issues in future research.

The ERB recognises three types of ethical review requirements:

- Full review, which requires the participation of all the ERB members, is warranted if a procedure or therapy of unknown effectiveness or efficacy is tested on people and/or if the research involves collecting body/tissue samples with hypothesis testing. Full review is needed, therefore, for all clinical trials and for some operational research projects.
- Expedited review, which requires the participation of two or three ERB members, is deemed sufficient if the research carries only minimal risks to human participants. Research in this category includes descriptive studies that involve monitoring and evaluation to test a new approach, social science research in health and health systems, and prevalence and incidence studies.
- Review exemption applies to routine programme implementation and assessment-related work.

Approval of research by the ERB is time limited—the study must start within one year. Investigators must inform the ERB about amendments to approved protocols, about the results of interim analyses that may lead to changes in protocol, and about early stopping of a trial and/or abandonment of a study. The ERB also asks to receive the final results of the research either as an internal report or as a published paper. However, the ERB has no direct oversight of research implementation and is often unable to judge whether the research results benefit the study population. Furthermore, not all research carried out by MSF is submitted to the ERB for review.

### A Framework for Ethics Review

During the first two years of its existence, the ERB used a framework derived from general guidelines on research ethics such as the Declaration of Helsinki [16], the Belmont report [17], and the Council for International Organizations of Medical Sciences guidelines [14] as a basis for discussion when members' opinions diverged. In 2003, in order to provide structured advice to field researchers and to facilitate standardised reviews, the ERB decided to adapt a draft framework for clinical research in the developing world developed by the National Institutes of Health in the United States [18]. This framework was tested by the ERB for 18 months to assess its utility and feasibility and, as a result, some of its benchmarks were changed or expanded. Since March 2005, the revised version of the framework has been used by the ERB (can be obtained from MSF on demand) and, as hoped, has standardised the review process and provided field research teams with valuable guidance on how to address ethical issues.

### Review Activity

Table 1 shows the workload of the ERB since its inception. In 2006–2008, the main themes of the protocols reviewed were HIV/AIDS (16), tuberculosis (12), and malaria (13). A further ten studies were concerned with tropical diseases such as leishmaniasis, cholera, Chagas disease, Buruli ulcer, Marburg fever, and kala-azar. The remaining protocols reviewed over this period focused on mental health, reproductive health, and nutrition.

**Table 1:** Number of protocols reviewed per year and status of research.

Number of Proposals/State of Research	2002	2003	2004	2005	2006	2007	2008
Number of proposals	5	8	6	3	14	34	23
Research articles published	2	6	2	1	4	8	0
Internal MSF reports produced	1	2	3	1	6	4	5
Research ongoing	0	0	0	0	2	19	18
Research cancelled/interrupted	2	0	1	1	2	3	0

Note: 11 proposals were submitted between January–March 2009.

As shown in Table 2, most studies reviewed in 2006–2008 were designed to either test a new intervention (diagnostic test or clinical procedure) or to assess the effectiveness of an intervention through a prospective descriptive study (49/70 protocols reviewed). Only five clinical trials, comparing the effectiveness of two or more treatment schemes, were submitted. On the other side of the research spectrum, a posteriori analysis of routinely collected data represented almost a quarter of the requested reviews.

**Table 2:** Type of research and approval process for studies submitted 2006–2008.

Approval Process	Research Category				
	Randomised Clinical Trial	Testing New Procedure (Laboratory Test, Intervention)	Prospective Descriptive Study	A Posteriori Analysis of Routinely Collected Data	Total
Direct approval	0	1	4	5	10
Approval after resubmission^a^	4	14	20	3	41
No approval	1 (abandoned)	4 (2 abandoned; 1 without approval, 1 pending)	2 (pending)	0	7
Advice before publication^b^	0	0	4	8	12
Total	5	19	30	16	70

a In most instances this means one cycle of resubmission; in a few cases, there were two cycles of resubmission.

b Studies that were not submitted to ethics review prior to implementation cannot be approved after the fact. However, the ERB has agreed to provide advice on ethical issues prior to publication.

## Some Challenging Ethical Issues

Since its inception, the ERB has faced several important ethical challenges. Some of these challenges have been resolved after extensive discussion at ERB meetings but others remain unresolved.

### Routine Data Monitoring

In MSF programmes, data are routinely collected as part of clinical practice without ethics approval. When these data are used a posteriori to test a research hypothesis, ethics review should be sought before doing the analysis, but as Table 2 shows, this has not always been the case. In its discussions, the ERB agreed that in most a posteriori studies, individual informed consent is not feasible or not deemed necessary. However, community consent may be necessary if there is the potential for harm to the community. Other ethical issues related to routine data monitoring that the ERB decided must be considered are local partnerships, the social value of the proposed research, and the possible benefits for the communities involved. In addition, the ERB decided that authorisation from the ministry of health concerned and/or a local health institution should always be requested before data are used retrospectively to test a research hypothesis. Box 1 provides an example of how the ERB dealt with a specific instance of retrospective data analysis.

**Box 1. Retrospective Analysis of Data from Patients with Kala-Azar**

Data were routinely collected during clinical practice in two settings in Africa and analysed a posteriori to assess treatment effectiveness and risk factors for relapse of kala-azar. The ERB approved this research because its social value is potentially high. No individual or community consent for the data analysis was deemed necessary in this context. However, local health authorities were informed and agreed to the research. Importantly, MSF continues to work in these settings and will be able to implement and advocate for a modification of treatment protocols if applicable, and patients and communities will be informed about the outcomes of the analysis.

### Emergency Research

In the past, MSF has often carried out research in emergency situations without first requesting ethics review. The ERB has then been asked retrospectively to review emergency research by providing advice on draft papers before their publication. Because emergency research is mostly carried out among highly vulnerable populations, such research may have serious implications for study participants [19],[20]. Consequently, the ERB and MSF recently agreed that to facilitate research in disaster situations, a “generic” research protocol could be submitted for ERB review and approval before the exact location of the disaster is known. Once the location is known, the details can be filled in and subjected to expedited review to allow the protocol to be applied in a specific setting. MSF took this approach, for example, when preparing a protocol for research into meningitis treatment before an expected outbreak actually occurred. Importantly, even if the MSF ERB grants “a priori” approval for emergency research, the ERB has specified that approval from the national authorities must always be sought before the research project is implemented.

### Qualitative Research

Investigations involving qualitative research methods are sometimes not considered research by MSF (and hence not submitted to ethics review), and the people involved in these studies are seen as “informants” rather than “research participants” [21]. In its discussions, the ERB decided, however, that ethical review might sometimes be necessary in qualitative research. For example, in one study of sexual violence, information was collected from affected women during in-depth interviews [22]. This type of interview carries a risk of psychologically harming people who have had traumatic experiences. Ethical review would help to assess the risk to individual participants. It would also examine the immediate value to the research community and ensure the quality of informed consent and information gathering.

### Community Involvement in Research

As well as the challenge of defining who or what constitutes a “community” [23], particularly in the humanitarian context where a community may be unstable and transient, a question raised in almost every proposal considered by the ERB is what qualifies as community engagement? One of the greatest ethical challenges facing the ERB and MSF is to find ways in which community participation can be ensured and enhanced while being realistic about time and resource constraints. This is a particularly large challenge because permission from state authorities and scientists is sometimes confused with genuine community engagement.

Ideally, a “functional” community body (e.g., village committees, community advisory boards) should be involved in each research project. This can be an existing body or one created for the specific purpose of the project. At a minimum, the community should be consulted during the planning stage of the research, should be consulted on an ad-hoc basis while the research is being done, and should be informed in a structured manner at the end of the research about the results. The ERB's insistence on involving the community early on in the research process has led to more explicit deliberation on this issue than previously (see Box 2 for a specific example).

**Box 2. Community Involvement in a Comparative Trial Examining the Efficacy and Safety of Three First-Line Treatments for Visceral Leishmaniasis in Africa**

The ERB found it difficult to judge from the initial proposal how collaboration with local communities would be developed and how the community's values, culture, traditions, and social practices would be taken into consideration in developing the research. The investigators replied: “MSF has been working in the study community for over 7 years. We have a long tradition of close collaboration with the local community in this project towards patient treatment. We have two staff members—one a religious leader, the other a member of the local popular committee—who have already been engaged and will continue in disseminating information of the study to the local community. With these direct links as well as through our other liaisons with the community (e.g.: health educators) we aim to clearly explain the reasons and possible long-term benefits of the study to community and religious leaders, village elders and the community at large. We understand that building a collaborative partnership with the local community is crucial for facilitating patient recruitment, treatment and follow up.”

One particular ethical issue discussed by the ERB that faces MSF is ensuring that communities understand the difference between receiving care and being involved in research. A physician's first goal is to help the patient; a researcher's first goal is to find an answer to a research question. As MSF does research in settings where the organisation is already present in the community and providing care, it can be difficult for potential study participants to understand the difference between receiving care and participating in research. Thus, it is important to engage the community as early as possible in designing the research, to have a dialogue throughout the study, and to communicate the results and implement change after the research is complete. In addition, the different ways of acting in regular programmes and in research must be clearly laid out and, where relevant, different contacts in the community (elders, leaders, and district health officials) should be established for operational purposes and for research purposes. MSF should also be careful not to “overuse” a community that is well engaged by doing research in that community on numerous occasions. Finally, where MSF is the sole care provider, it should be aware that the community may not feel able to refuse or criticise research, and must guard against that risk.

### Informed Consent

Obtaining quality informed consent was one of the major ethical challenges in most of the research proposals reviewed by the ERB. Examples of shortcomings include: incomplete information given to the participants about objectives, risks, adverse effects, and planned house visits; information too detailed and complicated; formulation of the text biased to induce a positive answer; overestimation of the benefit for participants and community; and lack of procedures to ensure that the information provided is understood.

As well as suggesting ways to improve the information provided, the ERB urges researchers to put more emphasis on the information exchange process between researchers and potential participants rather than on the formal consent form. For example, potential research participants should be given the opportunity to discuss their decision with their families, and alternative ways to record consent if individuals do not want to sign a consent form but are willing to participate in the proposed research should be sought. In some settings where MSF operates, people have declined to sign a consent form but are prepared to sign a register. In other settings, people have declined to sign anything but have given oral consent. In such circumstances, the ERB suggests that the researcher can keep a written record that the patient has been informed, understood, and accepted to participate, but declined to sign.

### Collecting and Using Tissue Samples

The collection, export, and analysis of tissues raises a host of ethical issues concerning the potential commoditisation and traffic of human identity and the exploitation of communities from which tissues have been taken [24]–[26]. Tissue samples are a precious commodity and extremely useful in the development of new diagnostic tests for rare diseases such as Ebola (see Box 3), Marburg fever, and sleeping sickness.

**Box 3. Tissue Samples Collected During an Ebola Outbreak**

MSF has repeatedly been involved in treatment of patients and infectious control during Ebola outbreaks. One major ethical issue that MSF has encountered during these outbreaks is the fact that other organisations sometimes take tissue samples from patients treated by MSF for further use without explicit consent. Ideally, at the very minimum, the results of any investigations done on these samples should be communicated to the patient and to the team in charge of their clinical management. Unfortunately, there have been instances where results have never been provided. In addition, it should also become standard practice that the patient is informed about the fate of his/her tissue sample (analysis and future use).

One proposed solution to the ethical issues associated with tissue sample collection and future use is to get prospective, one-time approval from study participants as part of the individual informed consent procedure and then to get approval from an ERB for any subsequent specific research that uses these tissue samples [27].

Since its inception, the MSF ERB has sought to create a tissue policy consistent with MSF's humanitarian mandate to remedy the current lack of guidance on the management of samples in international guidelines. Essential to this policy is a commitment to serve the beneficiaries of a humanitarian medical intervention, not the interests of third parties such as the developers of commercial tests. The ERB recommends that if MSF engages in research that involves the use of tissue samples (including export and/or storage for future use):

- Informed consent must be obtained, whenever possible, from the research participants on the intended use of the samples (but see Box 4).
- The use of samples taken by MSF and analysed in another laboratory should be regulated through a memorandum of understanding (future use, destruction, etc.).
- When samples are stored, the laboratory storing the sample should provide an annual report describing the fate and use of samples.
- Before future use of anonymised individual samples, community consent should be sought.

**Box 4. The ERB's Position on the Use of Tissue Samples without Informed Consent**

Investigators should be granted permission to use samples belonging to deceased, lost to follow-up, withdrawn, and completed follow-up participants only if:

1. The investigators omitted to obtain informed consent prospectively for the samples in good faith (i.e., the investigators' omission to obtain the informed consent for future sample use was not willful or deliberate);
2. The investigators have made reasonable and good faith attempts to trace those whose samples they seek to use (i.e., investigators must have made verifiable attempts to locate the relevant participants and they must produce documentation if requested that details the tracing process for each participant whose samples they seek to use);
3. The investigators have engaged with the host community on the issue and have received documented support from community representatives (such as a community advisory board);
4. The use of samples in the manner requested is not against local laws (the onus is on investigators to ascertain this and to notify the ERB accordingly);
5. There is a good scientific rationale for using such samples (the onus is on the investigators to show why they cannot use prospectively collected samples for their study);
6. The investigators/sponsor must clarify whether use/analysis of the samples could result in intellectual property/commercial implications. If so, they must outline a benefit-sharing plan or post-trial access plan with the local community. Such plan(s) should result from prospective engagement with the host community/community representatives and local authorities.

These recommendations have led to a much greater awareness on this issue in MSF and are now being included in the MSF research proposals sent to the ERB for review.

### Collaboration with For-Profit Entities

Sometimes MSF carries out operational research to field-test new technological devices. The results of this research could potentially benefit for-profit entities. A major ethical issue, therefore, is whether the benefits of the research will be shared with the study participants and community, either through profit sharing or through a guarantee of preferential access. Both these benefit-sharing approaches need to be negotiated before the research starts. In most instances preferential access is the easier and better option.

If MSF carries out research with a for-profit entity or research that could directly benefit a for-profit entity, the ERB has suggested the following guidelines:

- Informed consent must be obtained and must ensure that the research participant is fully informed about the potential commercial benefits of the research.
- Preferential access should be guaranteed through a contractual agreement between the company and the government of the country where the research takes place; if useful, this agreement should also involve MSF.
- Ethical considerations related to benefit sharing must be given due attention before the research starts and should include discussions with the communities involved.

As illustrated in Box 5, these recommendations have had some impact in the field. However, the development of a consistent MSF policy that includes these recommendations remains a challenge.

**Box 5. Testing the Effectiveness of a New Tuberculosis Detection Device**

A study had been designed to use sputum samples taken during routine clinical assessment from patients at high risk of multidrug-resistant tuberculosis to compare conventional culture methods with a new, more rapid tuberculosis culture and drug susceptibility device. The social value of the study was potentially high and the risk to research participants minimal. However, the results of the research could lead to commercial benefits. The investigators were not intending to seek informed consent and did not intend to inform the patient about the use of their samples in this study. The ERB pointed out that: “Sharing fairly the financial and other rewards of the research is a major ethical issue. This study uses an area of high tuberculosis burden to get the data needed to perfect a technology with commercial potential. The patients are unaware that their illness will eventually increase the profit of the corporation that produces the technology…There is a risk of fostering dependence on a superior technology without assuring affordability past a very narrow time horizon. MSF should thus insist on much stronger long term affordability. We would like to be assured of MSF's commitment to do so.” Consequently, a consent procedure was developed that explicitly informed patients about the further use of their sputum samples, about the partners involved in the research, and about potential commercial benefits. MSF also made a commitment to more proactive advocacy regarding access to the new test in the future.

### Making Research Benefits Available

MSF usually carries out research with the intention of delivering the results of the study to the community involved in the research. However, MSF may sometimes want to test an intervention that is too expensive at the outset of the research to be made immediately available to everyone who needs it. In view of the ERB, MSF can start such research if there are good reasons to expect a considerable price drop and if MSF initiates advocacy and lobbying efforts at the same time. This was the situation with the randomised controlled trials of artemisinin combination therapy for malaria that were largely undertaken in Africa and Asia and where price drops have since been achieved and easier-to-use formulations have become available. Box 6 illustrates how uncertainty about the affordability of an intervention can remain at the time of approval.

**Box 6. Testing a Nutritional Intervention That May Not Be Sustainable**

A proposed study of the effectiveness of a patented ready-to-use therapeutic food (RUTF) in catch-up growth of children after an acute episode of malaria in a hyperendemic area in Africa was considered by the ERB. The usefulness of providing supplementary food to children in convalescence was not questioned by the ERB. However, this RUTF is expensive and would have to be delivered on a broad scale in a severely resource-constrained environment. The ERB thus questioned the appropriateness of the research. MSF argued that an initial pilot study was necessary to assess the benefits of providing RUTF in convalescence since it could not ask for government commitment to the intervention without evidence that it works effectively. On a different level, MSF is currently campaigning to change international recommendations on early treatment and prevention strategies in malnutrition and is also working on price reductions of this specific RUTF as well as increasing the supply, possibly through local production, of other RUTFs.

A further issue is the commitment of MSF to make the intervention tested in the research available to the community [14],[28] for a certain time period. In view of the ERB, “reasonable availability” of an intervention tested means that MSF should commit to stay for a minimum of two years after the end of the research. If the organisation leaves, the intervention should be made available to the local population through other means (e.g., other international organisation or the country's ministry of health). However, the fact that MSF may leave earlier than anticipated due to organisational or political reasons and may not be able to engage local authorities in the provision of research benefits remains an ethical concern.

## Conclusions

The ERB instituted by MSF has been in place for seven years, and the need for independent ERB review of MSF research in addition to local ethics approval is now well accepted within the organisation. The MSF ERB often has a different perspective from that of academic institutions. In particular, it is more oriented towards programmatic relevance (feasibility issues) and is sensitive to vulnerable populations and equity issues.

The number and quality of research proposals reviewed by the ERB has increased considerably year on year. As well as scrutinising the ethical soundness of MSF research protocols, the ERB has also played an important role in launching an on-going debate on research ethics issues within MSF. The ERB recommendations have sensitised MSF researchers to ethical issues that they may have overlooked previously and have consequently changed practice. However, it will take more time to translate these recommendations into organisational policy.

International humanitarian organisations such as MSF will be faced with even more complex health problems in the future as the global environment changes. Research to devise and test new interventions will remain an important part of MSF's agenda, and will most probably increase. As this happens, a major concern will be to ensure that communities in which such research takes place are empowered to become true partners and that vulnerable individuals and groups are effectively protected [29]. The ethical oversight provided by the MSF ERB will be crucial in addressing these challenges.

## Abbreviations

ERB: ethics review board
MSF: Médecins Sans Frontières
RUTF: ready-to-use therapeutic food
